# Rapalogs can promote cancer cell stemness *in vitro* in a Galectin-1 and H-ras-dependent manner

**DOI:** 10.18632/oncotarget.17819

**Published:** 2017-05-11

**Authors:** Itziar M.D. Posada, Benoit Lectez, Mukund Sharma, Christina Oetken-Lindholm, Laxman Yetukuri, Yong Zhou, Tero Aittokallio, Daniel Abankwa

**Affiliations:** ^1^ Turku Center for Biotechnology, Åbo Akademi University, Tykistökatu 6B, Turku, Finland; ^2^ Department of Integrative Biology and Pharmacology, McGovern Medical School, University of Texas Health Science Center at Houston, Houston, Texas, United States of America; ^3^ Institute for Molecular Medicine Finland, FIMM, University of Helsinki, Helsinki, Finland; ^4^ Department of Mathematics and Statistics, University of Turku, Turku, Finland

**Keywords:** mTORC1, Ras, rapamycin, galectin, cancer stem cells

## Abstract

Currently several combination treatments of mTor- and Ras-pathway inhibitors are being tested in cancer therapy. While multiple feedback loops render these central signaling pathways robust, they complicate drug targeting.

Here, we describe a novel H-ras specific feedback, which leads to an inadvertent rapalog induced activation of tumorigenicity in Ras transformed cells. We find that rapalogs specifically increase nanoscale clustering (nanoclustering) of oncogenic H-ras but not K-ras on the plasma membrane. This increases H-ras signaling output, promotes mammosphere numbers in a H-ras-dependent manner and tumor growth *in ovo*. Surprisingly, also other FKBP12 binders, but not mTor-inhibitors, robustly decrease FKBP12 levels after prolonged (>2 days) exposure. This leads to an upregulation of the nanocluster scaffold galectin-1 (Gal-1), which is responsible for the rapamycin-induced increase in H-ras nanoclustering and signaling output. We provide evidence that Gal-1 promotes stemness features in tumorigenic cells. Therefore, it may be necessary to block inadvertent induction of stemness traits in H-ras transformed cells by specific Gal-1 inhibitors that abrogate its effect on H-ras nanocluster. On a more general level, our findings may add an important mechanistic explanation to the pleiotropic physiological effects that are observed with rapalogs.

## INTRODUCTION

Cancer stem cells (CSC) are critical for tumor seeding and growth [1]. They have been identified in a variety of cancers, typically with the help of surface markers, such as CD44+/CD24- for breast CSC [2]. Moreover, CSC can be enriched by culturing them as tumorospheres [3]. Due to the drug resistance of CSC, specific drugs are needed that so far did not follow the rational of targeted therapy.

Currently combinations of mTOR- and MAPK-pathway inhibitors are being tested for the treatment of cancer [4, 5, 6]. Both pathways operate downstream of Ras broadly driving cellular growth and cell division. Notably, a net overactivation of these pathways is observed in almost all types of cancer [7]. This has led to the development of an arsenal of kinase inhibitors against mTOR or PI3K, but also MAPK-pathway components such as Raf, Mek and Erk [4, 8]. Classically, mTORC1, but less mTORC2 was inhibited by rapamycin and analogues (called rapalogs). Rapamycin and its analogs bind to FK506-binding protein 12 (FKBP12) thus forming an inhibitory complex that allosterically blocks the activity of mTORC1 [9]. In addition, FKBP12 negatively regulates H-ras signaling. Being a prolylisomerase, it catalyzes isomerization of a C-terminal proline of H-ras, which facilitates H-ras depalmitoylation and decreases H-ras plasma membrane residence [10]. Therefore, FKBP12 inhibitors can acutely promote H-ras signaling output.

Hotspot mutations in codon 12, 13 and 61 render Ras constitutively active and make it a major driver in cancer [11]. Unfortunately, we still lack clinically approved inhibitors against either of the cancer associated Ras isoforms H-ras, N-ras and K-ras with the two splice isoforms 4A and 4B [8]. The focus is currently on K-ras4B (herafter K-ras) specific inhibitors, as K-ras is the most frequently mutated Ras isoform and was recently established as a specific driver of stemness in cancer cells and target of cancer stem cell drugs [12, 13]. This contrasted with H-ras, which established itself seemingly as a counterbalance of K-ras in cancer and non-transformed cells [12, 14].

While details for the different oncogenic qualities of H-ras and K-ras are still incompletely understood, it is well established that functional differences already emerge within the plasma membrane. Cancer associated Ras-isoforms H-ras, N-ras and K-ras are laterally segregated into nanoscale domains and display conformational differences in the membrane [15, 16]. Importantly, nanoscale oligomerisation of Ras into so called nanocluster correlates with effector recruitment efficiency and MAPK-signaling output [17, 18]. Galectin-1 (Gal-1) positively regulates GTP-H-ras- and negatively GTP-K-ras- nanocluster [19]. Furthermore, Gal-1 has a pro-angiogenic and pro-migratory effect in gliomas and melanomas [20]. In other cancer types, e.g. breast cancer, Gal-1 knockdown improved drug sensitivity [21]. Altogether, increased Gal-1 levels have been associated with more aggressive tumor progression. This led to the development of inhibitors that are typically directed against the carbohydrate binding activity of the protein [22].

Here, we show that rapalogs degrade their target FKBP12, which upregulates the H-ras specific nanocluster scaffold Gal-1, MAPK-signaling, stemness properties and tumor growth. Our work suggests that rapalog treatment should be combined with a new type of Gal-1 inhibitor that interferes with its ability to stabilize H-ras nanocluster.

## RESULTS

### Rapalogs increase H-ras nanoclustering and H-ras-dependent MAPK-signaling

We recently found that the commonly used protein synthesis inhibitor cycloheximide (CHX), was paradoxically able to increase the number of mammospheres and tumor growth in a H-ras dependent manner [23]. CHX exhibits a typically neglected polypharmacology, as it is not only an inhibitor of protein synthesis, but also of FKBP12, which in complex with rapalogs inhibits mTORC1.

**Figure 1:**
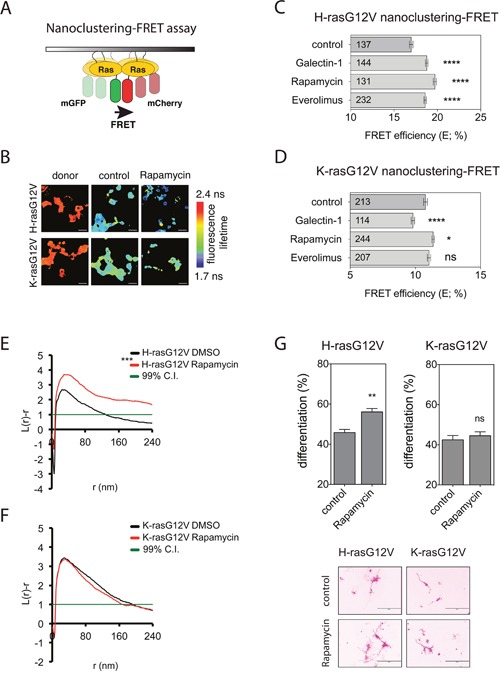
Rapalogs specifically activate H-ras nanoclustering and can drive PC12 cell differentiation **(A)** Schematic illustration of the nanoclustering-FRET assay. FRET is increased due to the formation of transiently immobile signaling complexes, which lead to nanoscale clustering of Ras in the plasma membrane. **(B)** Representative FLIM-FRET images of HEK cells expressing mGFP/mCherry-H-rasG12V or mGFP/mCherry-K-rasG12V FRET pairs. Image color look-up table on the right shows fluorescence lifetimes, with low lifetimes indicating high FRET and high lifetimes indicating low FRET. Scale bar in the images represents approximately 20 μm. **(C and D)** Nanoclustering-FRET analysis in HEK cells co-expressing mGFP- and mCherry-tagged **(C)** H-rasG12V or **(D)** K-rasG12V. Cells were treated for 24 h with DMSO control, 0.5 μM rapamycin or 2 μM everolimus. In addition, co-expression of FRET pairs with Galectin-1 was used for comparison. The numbers in the bars indicate the number of analyzed cells (mean ± SEM, n=3). **(E and F)** Electron microscopic nanoclustering analysis of BHK cells expressing mGFP-tagged **(E)** H-rasG12V, **(F)** K-rasG12V with or without 0.5 μM of rapamycin. Intact apical plasma membrane sheets were immunolabeled with 4.5 nm gold nanoparticles coupled to anti-GFP antibody. The spatial distribution of gold particles was evaluated using univariate K-function, where L(r)–r values indicate the extent of nanoclustering as a function of the length scale, r, in nm. At least 15 images were analysed for each condition. Statistical significance between different conditions was evaluated using bootstrap tests. Averaged curves are shown for each condition. **(G)** PC12 cells transiently transfected with (*left*) mGFP-H-rasG12V or (*right*) mGFP-K-rasG12V were incubated with DMSO control or 0.5 μM rapamycin. After 72 h, GFP-positive cells were scored for neurites. Results (*top*) are plotted as percent of cells (mean ± SEM, *n* = 4) with neurite outgrowth >1.5 times the diameter of the cell body. Representative images of cells (*bottom*) scored for neurites are shown. Bar represents 200 μm.

We therefore tested, whether rapalogs could phenocopy the observations made with CHX. Given the increasing evidence that H-ras- and K-ras-isoforms have distinct, if not partly antagonistic functions [12, 14, 24], we employed a FRET-assay, which analyses the densely packed, active Ras proteins in isoform-specific signaling nanocluster in the membrane [25, 26] (Figure 1A, 1B). Treatment of HEK293-EBNA (hereafter HEK) cells with rapalogs increased specifically H-rasG12V nanoclustering-FRET to a similar level as the well-established nanocluster scaffold Gal-1 [19] (Figure 1C, 1D). While Gal-1 significantly decreased K-rasG12V nanoclustering-FRET without altering K-rasG12V subcellular distribution (Figure 1D and Supplementary Figure 1A), treatment with rapalogs had little effect on K-rasG12V nanoclustering-FRET in HEK cells (Figure 1D).

These effects on the nanoscale organization of Ras were confirmed by analyzing the point-pattern distribution of immuno-gold labeled Ras on plasma membrane rip-offs from BHK cells. Rapamycin treatment significantly increased the L(r)-r value of H-rasG12V (Figure 1E), indicating upmodulated nanoclustering, while having no effect on K-rasG12V nanoclustering (Figure 1F).

Consistent with a strong activation of Ras signaling by augmented nanoclustering, rapamycin increased pErk and pAkt levels similar to CHX-treatment. (Supplementary Figure 1B).

Activation of Ras−MAPK-signalling is known to induce differentiation of rat adrenal pheochromocytoma (PC12) cells, which is visible by neurite outgrowth. This assay is therefore reporting on MAPK-activity [27]. Accordingly, expression of GFP-H-rasG12V or K-rasG12V induced differentiation of PC12 cells, as can be seen by neurite formation (Figure 1G). Previously, others and we have shown that H-rasG12V-driven PC12 cell differentiation was increased by treatments that increased H-ras nanoclustering, such as Gal-1 expression [28] or CHX treatment [23]. In line with the H-ras-specific effect of rapamycin on nanoclustering upon rapamycin treatment (Figure 1C, 1D), only H-rasG12V transfected PC12 cells showed an increased differentiation with rapamycin as compared to the non-treated control, while K-rasG12V transfected PC12 cells did not (Figure 1G).

Finally, in agreement with a previous report, which showed that everolimus could increase MAPK-signaling in a PI3K-dependent manner [29], both pharmacological inhibition of PI3K activity by wortmannin (Supplementary Figure 1C), as well as knockdown of p110α abolished the rapamycin-induced effect on H-ras nanoclustering-FRET (Supplementary Figure 1D, 1E).

Thus rapalogs increase specifically active H-ras-nanocluster and signaling output in a PI3K-dependent manner.

### Rapalogs promote mammosphere formation and *in ovo* tumor growth in a H-ras-dependent manner

**Figure 2:**
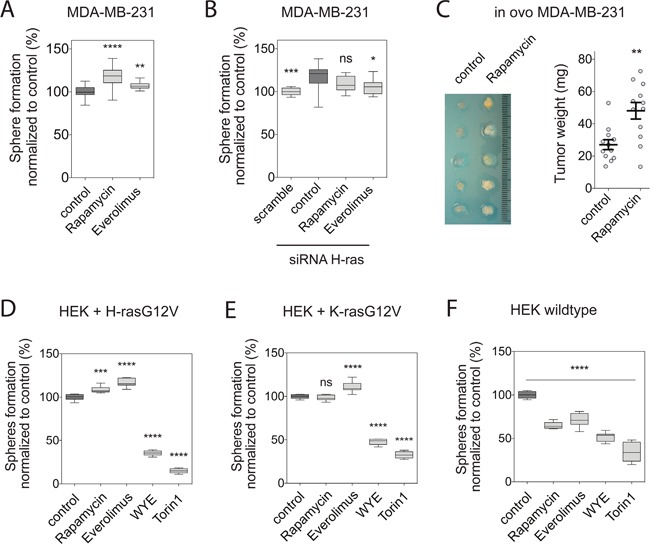
Rapalogs promote tumorigenicity H-ras dependently **(A and B)** Mammosphere formation efficiency of MDA-MB-231 cells grown in non-adherent conditions. Mammospheres, **(A)** not transfected or **(B)** transfected with scrambled or H-ras siRNA, were allowed to form for 6 days and treated for additional 3 days with either DMSO control, 0.5 μM rapamycin or 2 μM everolimus (n=3). **(C)** *Left*, representative images oftumors derived from MDA-MB-231 cells after transplantation onto chick embryo CAM and treated for 4 days with either DMSO control or 0.5 μM rapamycin. *Right*, the tumor weight was calculated from 12 tumors from four independent experiments (mean ± SEM). **(D to F)** Sphere-forming efficiency of **(D)** HEK transiently expressing mGFP-H-rasG12V or **(E)** mGFP-K-rasG12V, or **(F)** wildtype HEK cells grown in non-adherent conditions. Spheres were allowed to form for 6 days and treated for additional 3 days with either DMSO control, 0.5 μM rapamycin, 2 μM everolimus, 2 μM WYE-125132 or 0.25 μM torin 1 (n=3). **(A-F)** Statistical comparisons are done with the dark-grey highlighted control samples. Notice that in **(B)** this is the siRNA H-ras treated sample.

As previously observed with CHX [23], rapalogs significantly increased MDA-MB-231 mammosphere formation in a H-ras dependent manner (Figure 2A, 2B). Note, that H-ras knockdown alone increased sphere formation relative to the scramble control, consistent with its negative effect on K-ras nanoscale organization and signaling, which is mediated by perturbation of the phosphatidylserine distribution by oncogenic H-ras [24]. This sharply contrasted with the effect of mTor kinase inhibitors, which highly significantly blocked sphere formation (Supplementary Figure 2A). In agreement with the increased mammosphere numbers, rapamycin increased tumor growth when MDA-MB-231 cells were xenografted onto the chorioallantoic membrane of chick embryos for *in ovo* tumor formation [30] (Figure 2C).

Interestingly, MDA-MB-231 cells carry a K-rasG13D mutation, suggesting that the pro-tumorigenic effect is communicated by endogenous, wildtype (wt) H-ras in these cells. However, neither rapamycin, nor everolimus did have a positive effect on MCF7 sphere numbers, which are wildtype for Ras, but decreased mammospheres as TOR-kinase inhibitors did (Supplementary Figure 2B). By contrast, everolimus significantly increased sphere numbers in H-rasG12D-mutated Hs578T (Supplementary Figure 2C).

In order to understand, whether the observed sphere growth modulation depends on the expression of oncogenic Ras, we employed spheres derived from RasG12V-expressing HEK cells [31]. This model reproduced the significantly higher potential of oncogenic K-ras to increase sphere numbers, as compared to oncogenic H-ras (Supplementary Figure 2D, 2E) [12].

Intriguingly, HEK cells that were transfected either with H-rasG12V or K-rasG12V recapitulated the rapalog-induced increase in sphere numbers that was observed in breast cancer cell lines, while remaining sensitive to mTOR inhibitors (Figure 2D, 2E). By contrast, wt HEK cell derived spheres were sensitive to all of these inhibitors (Figure 2F), as observed for MCF7 cells (Supplementary Figure 2B).

Therefore rapalogs have the potential to increase the sphere-forming capacity of tumorigenic cells that were reprogrammed with oncogenic Ras.

### Reciprocal regulation of FKBP12 and galectin-1 links FKBP12-level modulation to H-ras nanoclustering

**Figure 3:**
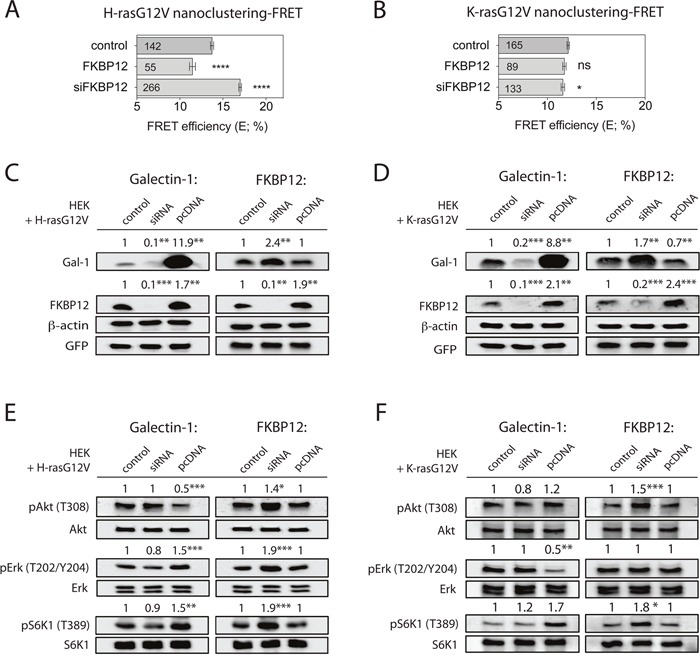
A galectin-1-dependent FKBP12 rescue-loop is activated upon rapalog-induced FKBP12 downmodulation **(A and B)** Nanoclustering-FRET analysis in FKBP12 knockdown/overexpressing HEK cells co-expressing mGFP- and mCherry-tagged **(A)** H-rasG12V or **(B)** K-rasG12V. The numbers in the bars indicate the number of analyzed cells (mean ± SEM, n=4). **(C and D)** Western blot analysis in HEK cells transfected with the indicated FKBP12 or Gal-1 siRNAs, and in addition expressing **(C)** mGFP-H-rasG12V or **(D)** mGFP-K-rasG12V. Control is transfected with empty vector, while pcDNA marks exogenous expression of protein indicated on top of the blots. Numbers indicate β-actin normalized protein levels (n=4). **(E and F)** Western blot analysis of Ras and mTORC1 signaling in HEK cells expressing **(E)** mGFP-H-rasG12V or **(F)** mGFP-K-rasG12V under indicated FKBP12 or Gal-1 manipulations as in **(C and D)**. Numbers indicate the ratio of phosphorylated to respective total protein levels (n=3).

We next asked, whether downmodulation of FKBP12 levels could phenocopy the nanoclustering effects of rapalogs. Indeed, this was seen after knockdown of FKBP12, which specifically increased H-ras nanoclustering-FRET (Figure 3A), but had only a very small effect on K-ras nanoclustering-FRET (Figure 3B). Conversely, increased expression of FKBP12 downmodulated specifically H-rasG12V nanoclustering-FRET (Figure 3A) [10], but had no effect on K-ras nanoclustering-FRET (Figure 3B).

So far, we only know of the nanocluster scaffold Gal-1 as a positive modulator specifically of H-ras nanoclustering. Consistent with an involvement of Gal-1, knockdown of Gal-1 significantly attenuated the positive effect of rapamycin on H-ras nanoclustering (Supplementary Figure 3A). We therefore examined the expression of Gal-1 upon FKBP12-level modulation in wt HEK or HEK expressing oncogenic Ras. Intriguingly, knockdown of FKBP12 significantly upregulated Gal-1 levels, while FKBP12 overexpression had no or just a small effect on Gal-1 expression (Figure 3C, 3D and Supplementary Figure 3B, 3C).

Conversely, we found that the knockdown of Gal-1 strongly downmodulated FKBP12 levels, while high Gal-1 overexpression increased FKBP12 levels in wt and Ras-expressing HEK cells (Figure 3C, 3D and Supplementary Figure 3B, 3C). Thus each of these four expression manipulations resulted in a specific set of Gal-1- and FKBP12-level combinations (Table 1).

**Table 1:** Summary of major experimental results

	H-rasG12V						K-rasG12V				
	Gal-1	FKBP12	nano-clustering	pERK	pAKT	pS6K	nano-clustering	pERK	pAKT	pS6K	sphereformation
Gal-1	+	+	+	+	–	+	–	–	0	+	+ *
siFKBP12	+	–	+	+	+	+	–	0	+	+	+
siGal-1	–	–	0	0	0	0	n.d.	0	0	0	–
FKBP12	0^#^	+	–	0	0	0	0	0	0	0	–

Changes relative to control conditions are denoted with + for increased and – for decreased parameters; 0 means no significant change and n.d. not determined. Left column shows manipulations: overexpression of Gal-1 (Gal-1), knockdown of FKBP12 (siFKBP12), knockdown of Gal-1 (siGal-1) and overexpression of FKBP12 (FKBP12). Sphere formation was analyzed in Hs578T, MCF7, MDA-MB-231 and HEK cells with and without oncogenic H-ras or K-ras expression. The symbol # indicates that a decrease in Gal-1 expression was observed only in oncogenic K-ras expressing cells. The symbol * indicates that sphere growth was not observed in oncogenic K-ras expressing cells.

In agreement with the nanoclustering changes (Figure 1C, 3A), Gal-1 overexpression and FKBP12 knockdown led to a similar increase in pErk and pS6K1 levels in H-rasG12V transfected HEK cells (Figure 3E). However, as observed by others and us [25, 32], high levels of Gal-1 decreased pAkt, while FKBP12 knockdown increased it (Figure 3E). No significant changes of the above phosphoproteins were observed when Gal-1 was knocked down or FKBP12 was overexpressed (Figure 3E).

In line with the negative effect of Gal-1 on K-rasG12V-nanoclustering, pErk levels were significantly decreased, while pS6K1 was somewhat increased and pAkt remained unchanged in Gal-1 overexpressing K-rasG12V-HEK cells (Figure 3F). By contrast, phosphorylation of pErk remained unchanged, while pAkt and pS6K1 were significantly increased in those cells after FKBP12 knockdown (Table 1).

These data imply that downmodulation of FKBP12 increases H-ras nanoclustering by the associated Gal-1 upregulation.

### Galectin-1 and FKBP12 level modulation affect mammosphere formation

**Figure 4:**
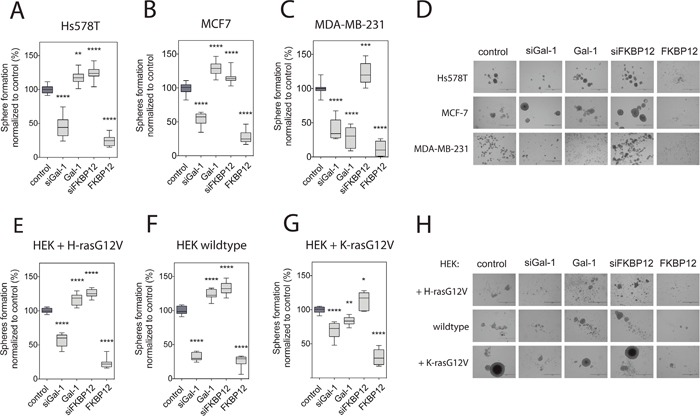
Galectin-1- and FKBP12-level modulation affects sphere formation consistent with their effects on Ras nanoclustering **(A to C)** Effect of Gal-1 and FKBP12 level modulation on Hs578T **(A)**, MCF7 **(B)** and MDA-MB-231 **(C)** cell sphere formation. Cells were transfected with the indicated expression constructs or siRNA (si pre-fix), and spheres were allowed to form for 9 days (n=4). **(D)** Representative images of cancer cell spheres from **(A-C)**. Bar in the images represents 1000 μm. **(E to G)** Effect of Gal-1 and FKBP12 level modulation on sphere formation of wildtype or **(E)**, H-rasG12V- **(F)** or K-rasG12V- **(G)** expressing HEK cells. Cells were transfected with the indicated expression constructs or siRNA (si pre-fix), and spheres were allowed to form for 9 days (n=3). **(H)** Representative images of HEK spheres from **(E-G)**. Bar in the images represents 1000 μm.

Given the similarities of the rapalog- and FKBP12-induced nanoclustering responses, we hypothesized that loss of FKBP12 may increase mammospheres. Indeed, knockdown of FKBP12 increased sphere formation in H-rasG12D mutated Hs578T (Figure 4A, 4D). Interestingly, the same was not only observed with Ras wt MCF7 spheres (Figure 4B, 4D), but also in K-rasG13D mutant MDA-MB-231 spheres (Figure 4C, 4D). By contrast, overexpression of FKBP12 basically abrogated mammosphere formation from all of these three cell lines (Figure 4A-4D). Hence, FKBP12 upregulation may have effects that do not correlate with the Erk- and Akt-signaling (Figure 3E, 3F), but with its effect on H-ras nanoclustering-FRET (Figure 3A) [10].

Low FKBP12 levels also lead to high Gal-1 levels (Supplementary Figure 3C), suggesting that Gal-1 could be responsible for the increased sphere growth with decreased FKBP12 levels. Indeed, Gal-1 overexpression phenocopied the sphere promoting effect of the FKBP12 knockdown in Hs578T and MCF7 cells (Figure 4A, 4B, 4D), but not in MDA-MB-231 cells (Figure 4C, 4D). In fact, the decreased sphere growth in K-ras-mutant MDA-MB-231 cells was consistent with the negative effect of Gal-1 on active K-ras nanoclustering (Figure 1B, 1D).

In order to understand how much the observed sphere growth modulation depends on the Ras-mutation status as compared to other mutations, we employed again the HEK-cell derived sphere model. Comparison of Figure 4A-4D with Figure 4E-4H clearly demonstrates that the HEK-spheres phenocopied the treatment response of the breast cancer cell lines, if both expressed the same oncogenic Ras-isoform (Figure 4A, 4E and 4C, 4G). These data suggest that Gal-1 promotes sphere growth in Ras wt and oncogenic H-ras transformed cells.

### Gene expression analysis suggests a galectin-1-associated stemness program

In order to understand what genetic characteristics are responsible for the sphere growth promotion by Gal-1 overexpression or FKBP12 knockdown, we analyzed the expression of genes that we previously associated with a K-ras directed CSC-drug response in HEK cells with the respective manipulations [13].

**Figure 5:**
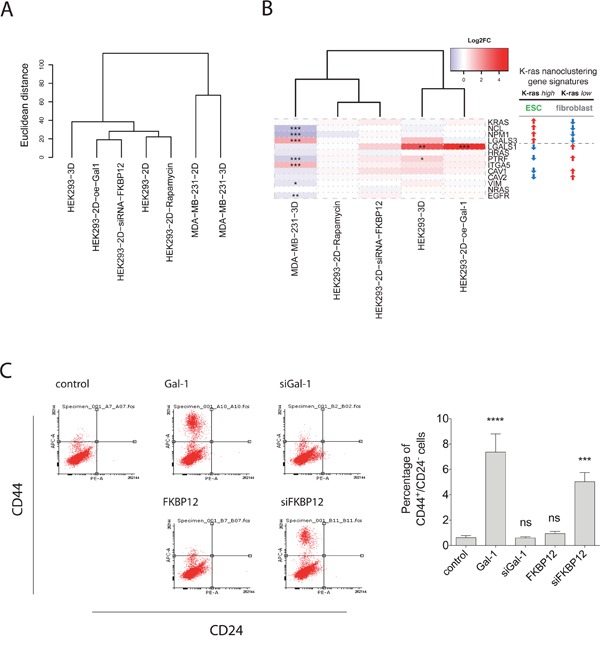
Gene expression analysis with RNA-seq supports stemness promoting activity of galectin-1 **(A)** Hierarchical clustering of samples using genome-wide RNA-seq data. Replicate samples (n=3) in each group are averaged and Euclidean distance is used as distance metric. **(B)** Heatmap of fold-changes (log2 scale) comparing each treatment group vs. 2D cultured control group for the genes of the K-ras nanoclustering associated gene signature. Right table shows the two-types of identified gene regulations of which the ESC-like type was predictive for K-ras directed CSC-drug response (10). * FDR < 0.05; ** FDR < 0.01; *** FDR < 0.001. **(C)** *Left*, CD44/CD24 FACS profiles are shown for HEK after FKBP12 or Gal-1 manipulation. *Right*, shown is the average percentage of CD44^+^/CD24^−^ HEK cells. Error bars denote the SEM from three independent experiments performed in duplicate.

This analysis revealed a similar overall gene expression response of HEK cells with overexpressed Gal-1 or downmodulated FBKP12 (Figure 5A). Intriguingly, the Gal-1 gene (LGALS1) was also significantly increased in HEK cells cultured as spheres (3D), as compared to those grown on plastic (2D) (Figure 5B).

Consistently, we could show that Gal-1 overexpression and FKBP12 knockdown in HEK cells upregulated the stemness marker CD44, thus establishing a CD44+/CD24- population which is also known to form aggressive tumors (Figure 5C) [31]. Also another gene, PTRF, that we previously associated with a more fibroblast-like [13], as compared to embryonic stem cell (ESC)-like gene signature was significantly increased in 3D-HEK (Figure 5B). However, none of this was observed for K-ras mutated MDA-MB-231 cells grown in 3D vs. 2D (Figure 5A, 5B).

We therefore tentatively conclude that Gal-1 is associated with a different, possibly opposite H-ras-driven stemness program (fibroblast-like), than the K-ras associated ESC-like program that we have previously described [13].

### Rapalogs induce downmodulation of FKBP12 thus increasing galectin-1 expression

The above results suggest that upregulation of Gal-1 after loss of FKBP12 promotes stemness in a H-ras dependent manner. We therefore scrutinized, the connection of the rapalog and FKBP12 knockdown effect.

**Figure 6:**
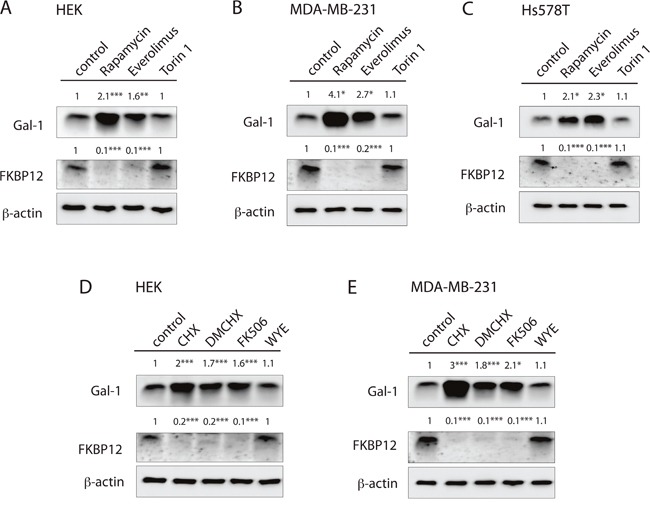
FKBP12-binding compounds stimulate FKBP12 downmodulation and couple Gal-1 induction **(A to E)** Western blot analysis of Gal-1 and FKBP12 protein levels in **(A, D)** HEK, **(B, E)** MDA-MB-231 and **(C)** Hs578T cells upon FKBP12 knockdown or treatment with 0.5 μM rapamycin, 2 μM everolimus, 0.25 μM torin 1, 0.18 μM CHX, 10 μM DM-CHX, 0.5 μM FK506 or 2 μM WYE -125132. Cells were treated for 48 h. Numbers indicate β-actin normalized protein levels (n=3).

Intriguingly, a loss of FKBP12 expression was observed in several cell lines with all FKBP12 binders, such as FK506 and a derivative of CHX, DM-CHX, which does not block protein synthesis [33], as well as with rapalogs (Figure 6A-6E). By contrast, mTor-kinase inhibitors torin 1 or WYE-125132 did not change FKBP12 levels (Figure 6A-6E).

**Figure 7:**
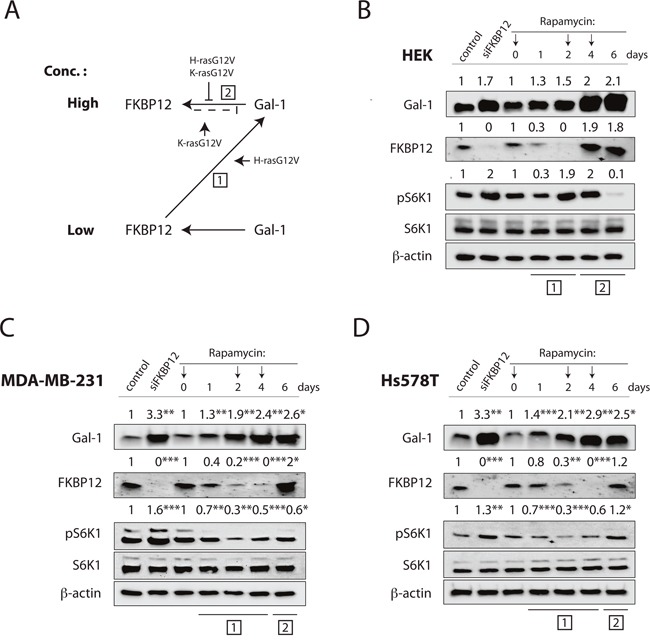
Rapamycin induces downmodulation of FKBP12 thus increasing galectin-1 expression **(A)** Proposed regulation scheme for Gal-1-dependent FKBP12-rescue loop. Low and High denotes concentration of proteins in the cell. Boxed numbers refer to stages in **(B, C and D)**. Small arrows and block lines indicate modulators. **(B to D)** Western blot analysis of Gal-1 and FKBP12 protein levels in **(B)** HEK, **(C)** MDA-MB-231 and **(D)** Hs578T cells upon FKBP12 knockdown or rapamycin treatment for 6 days. Numbers indicate β-actin normalized protein levels. pS6K1 is shown as a marker of mTorC1 activity, and numbers indicate the ratio of phosphorylated to respective total protein levels (n=3). Arrows on top indicate when rapamycin treatment was refreshed. Boxed numbers on bottom relate to the steps in **(A)**.

A detailed analysis of the protein level changes after Gal-1- and FKBP12-level manipulations (Supplementary Figure 3C) revealed a Ras-isoform modulated Gal-1 mediated ‘rescue loop’ of FKBP12 expression. Thus FKBP12 levels are Gal-1 dependently reinduced, if they fall low (Figure 7A). Notably, the level of FKBP12 induction by Gal-1 was significantly lower in HEK cells overexpressing H-rasG12V or K-rasG12V, while the induction of Gal-1 by the knockdown of FKBP12 was somewhat increased by H-rasG12V but not K-rasG12V (Supplementary Figure 3C). This indicates that in the presence of oncogenic Ras, FKBP12 expression is relatively less induced, if Gal-1 levels are high (Figure 7A, [2]). More importantly, Gal-1 levels seem to be relatively higher induced with low FKBP12 levels only in H-rasG12V expressing cells (Figure 7A, [1]). Therefore, in particular with oncogenic H-ras, higher Gal-1 levels and lower FKBP12 levels are promoted, overall supporting the H-ras stemness state.

This reciprocal regulation of FKBP12 and Gal-1 had interesting consequences, if tumorigenic cells were treated with rapamycin over the course of several days. Within two days after rapamycin treatment of HEK cells, FKBP12 levels decreased, while Gal-1 levels concomitantly increased (Figure 7B). Importantly, after FKBP12 is lost on day 2, high Gal-1 levels were followed by FKBP12 reexpression (Figure 7B).

Importantly, very similar observations were made when HEK cells were grown as spheres (Supplementary Figure 4A), supporting the significance of this reciprocal regulation also in sphere cultures. Basically the same occurred in MDA-MB-231 and Hs578T cells (Figure 7C, 7D), where however FKBP12 levels (and Gal-1 levels) are higher than in HEK cells to begin with (Supplementary Figure 4B). As a consequence FKBP12 expression is lost only on day 4. This rapamycin treatment induced FKBP12 degradation has therefore important consequences, as it promotes sphere growth, as long as Gal-1 levels are elevated (Figure 7A, [1]) and mTORC1 activity is not blocked (Figure 7B, day 6). Due to the reciprocal regulation of FKBP12 and Gal-1, and depending on the actual levels of these proteins, transient periods of sphere growth promotion may occur in a cyclic manner.

## DISCUSSION

The nested mTOR- and Ras-pathways are of critical importance to maintain tumor growth. Failure of mTORC1 inhibitors to efficiently kill cancer cells and the existence of multiple feedback loops require combinations with inhibitors, such as against the Ras/MAPK-pathway [5]. However, given that K-ras and H-ras appear to have different roles to promote stemness also in cancer cells [12, 14], it is critical to understand what the exact contributions from these individual Ras isoforms are in cancer.

**Figure 8:**
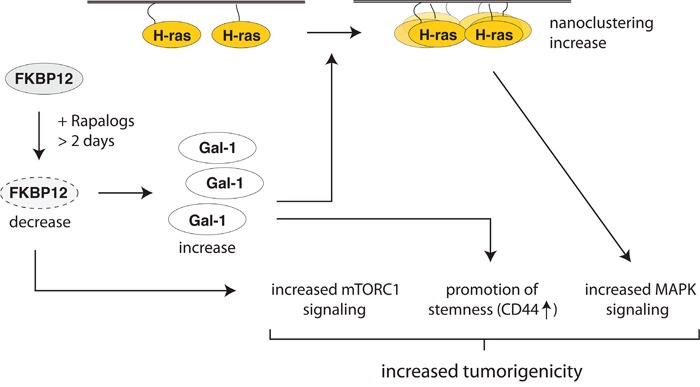
Summary scheme illustrating how rapalogs affect stemness and tumorigenicity Prolonged rapalog treatment decreases FKBP12 levels (dotted lines), which leads to an increase of Gal-1 expression and de-repression of mTORC1 signaling. Gal-1 stabilizes nanocluster of active H-ras, thus promoting MAPK-signaling. In addition Gal-1 stimulates other stemness features, such as CD44-expression. Altogether, these changes may increase tumorigenicity in certain situations.

We found that prolonged exposure of tumorigenic cells with rapalogs decreases FKBP12 levels (Figure 6), which potentially leads to hyperactivation of the mTORC1-pathway (Figure 3E, 3F). We are intrigued by the finding that a number FKBP12 binders degrade their target in several cell lines (Figure 6). Considering that rapamycin has been studied for many years, it is surprising that this has not been described before. It remains to be shown, whether this also applies to other FKBPs, such as FKBP51 and FKBP52 [34] and what the specific consequences would be. Most importantly however, loss of FKBP12 induces high Gal-1 levels, which increase specifically H-ras nanoclustering and thus MAPK-signaling output. Our data suggest that this could at least transiently increase tumor growth after rapalog exposure (Figure 8).

Similar to the FKBP12-associated mechanism described by Mark Philips group, our mechanism would promote H-ras signaling output. However, in Ahearn et al. acute inhibition of FKBP12 increases plasma membrane residence of H-ras [10]. By contrast, we observed FKBP12 degradation induced upregulation of Gal-1, which creates a delay of 2-4 days, while also lasting for 1-2 days. We consider the longer time frame of activity more relevant for cellular differentiation changes and establishment of stemness features. In support of this, it was recently observed that rapamycin induces the transcription of stemness factors such as Oct4, Sox2 and Nanog, while preventing replicative senescence in fibroblasts [35].

Comparison of sphere-formation data after rapalog treatment and knockdown of FKBP12 suggest that increased sphere growth after rapalog treatment requires expression of oncogenic Ras to sustain higher mTorC1 activity and probably also to support the induction of higher Gal-1 levels. Moreover, Gal-1 knockdown data from oncogenic K-ras expressing cells show that the loss of Gal-1 that is leading to a loss of FKBP12, is not promoting sphere growth as the loss of FKBP12 alone (which also increases Gal-1). This suggests that increased Gal-1 levels promote sphere growth of K-ras transformed cells only in the absence of FKBP12. These results also explain, how Gal-1 can execute paradoxical, opposite actions in cancer cells, which would depend on the expressed oncogenic Ras isoform and FKBP12 levels. However, most significantly in terms of new CSC-drug target identification, knockdown of Gal-1 or overexpression of FKBP12 always reduced sphere formation, irrespective of which oncogenic Ras was expressed (Figure 4A-4H).

We propose that interference with the H-ras nanocluster-promoting activity of Gal-1 may present an important target in this context. This may require novel inhibitors against Gal-1 that interfere with its activity to complex in particular with the Raf effectors; an interaction which is required for its nanocluster promoting activity [19]. Interestingly, the Gal-1 inhibitor OTX008, which is not a classical carbohydrate binding region competitor, was recently shown to synergize with rapamycin to block tumor growth [36]. Intriguingly, an OTX008 related compound binds at a Gal-1 site that may allosterically affect its binding to Raf-effectors or Gal-1 dimerization and hence its nanocluster scaffolding activity [37]. More generally, our work suggests that rapalog treatment should be combined specifically with H-ras signaling inhibition to prevent possibly detrimental shifts in MAPK- and Akt-signaling with consequences for stemness induction.

Given the transient nature of the rapalog induced FKBP12 degradation (due to the rescue-loop) and the dependence of the effect on cellular FKBP12 levels, as well as on the efficacy of FKBP12 degradation, it is obvious that current use of rapalogs does not massively lead to problems in the clinic. It therefore remains to be seen, whether rapalogs more frequently increase the cancer cell stemness-associated metastatic risk, as recently observed for everolimus [38].

## MATERIALS AND METHODS

### Materials

Reagents were obtained from the following sources. Antibodies to phospho-T202/Y204 Erk (catalogue no. 9101), Erk (no. 9102), Akt (no. 9272), phospho-T389 S6K1 (no. 9234), S6K1 (no. 9202), phospho-S235/S236 S6 (no. 2211), S6 (no. 2217) and PI3K p110α (no. 4255) were from Cell Signaling Technologies (Danvers, MA, USA); phospho-T308 Akt (no. MAB7419) from R&D Systems (Wiesbaden, Germany); FKBP12 (no. 610808) from BD Biosciences (San José, CA, USA); galectin-1 (no. 500-P210) from Peprotech (Hamburg, Germany); the secondary anti-mouse (no. sc-2954) and anti-rabbit (no. sc-2004) antibodies were from Santa Cruz (Paso Robles, CA, USA); GFP (no. 3999-100) was from Bio-Vision (Milpitas, CA, USA) and β-actin (no. A1978) from Sigma (Sigma-Aldrich, Helsinki, Finland). The compounds everolimus and WYE-125132 were from SelleckChem (Rungsted, Denmark), rapamycin was from Thermo Fisher Scientific (Grand Island, NY, USA), torin 1 was from Tocris Bioscience (Bristol, UK), cycloheximide was from Acros Organics (Thermo Fisher Scientific), wortmannin was from Cell Signaling Technologies, FK506 and compactin were from Sigma. 50x B27 supplement, horse serum, Opti-MEM and transfection reagents Lipofectamine 3000 and RNAiMAX were from Thermo Fisher Scientific; Transfection reagent JetPrime was from Polyplus (Illkrich-Graffenstaden, France); Dulbecco's Modified Eagle Medium (DMEM), Roswell Park Memorial Institute (RPMI), EGF and FGF were from Sigma.

### Cell culture

Human Embryonic Kidney 293-EBNA (HEK), BHK21, MCF7 and Hs578T cells were cultured in DMEM (Sigma-Aldrich, Helsinki, Finland). MDA-MB-231 cells were cultured in RPMI. Media were supplemented with 10% fetal bovine serum and 1% L-glutamine. Rat adrenal pheochromocytoma (PC12) cells were cultured on plates coated with 50 μg/mL of rat tail collagen I (Gibco) in Roswell Park Memorial Institute (RPMI)-1640 medium (Invitrogen) supplemented with 10% horse serum, 5% FBS, 1% L-glutamine, 100 U/mL penicillin, and 100 μg/mL streptomycin. All cells were incubated at 37°C with 5% CO_2_. They were grown typically to a confluency of 80% and subcultured every 2–3 days. Transfections were performed with JetPRIME (Polyplus, Illkrich-Graffenstaden, France) unless otherwise stated.

### DNA constructs and siRNA

Plasmids pmGFP-H-rasG12V, pmGFP-K-rasG12V, pcDNA3-Gal-1 were described before [15, 39, 40]. PmCherry-RasG12V constructs were generated by replacing mGFP from pmGFP-RasG12V with mCherry from pmCherry-C1 vector (Clontech Laboratories Inc., Mountain View, CA, USA) using NheI and BsrGI restriction sites [13]. FKBP12 was amplified from pOPINE-eGFP-FKBP12 using the following couple of primers: forward 5´-GACCAAGCTTACC ATGTCTAGAGGAGTG-3´ and reverse 5´-GCCAGAATTCTTAATAACT AGTTTCCAG-3´. The PCR product was purified and cloned into pcDNA3.1 vector using HindIII and EcoRI restriction sites. Final construct was verified by DNA sequencing (GATC, Köln, Germany).

The human gene directed siRNA catalogue numbers are as follows: scrambled siRNA (D-001810-10-20), siRNA directed against FKBP12 (L-009494-00) and PI3K p110α (L-003018-00) were from Dharmacon; siRNA directed against Galectin-1 (GS3956) was from Qiagen. SiRNA directed against H-ras was a kind gift of Dr. Jukka Westermarck [41]. For siRNA transfection JetPrime reagent was used, unless otherwise specified.

### FRET sample preparation

#### Compound treatments

HEK cells were seeded on a 6-well plate with glass coverslips, and transfected with the donor alone (mGFP-tagged Ras constructs) in control samples, or together with the acceptor using JetPRIME according to the manufacturer´s instructions. Acceptors were mCherry-tagged Ras constructs (mGFP-: mCherry-plasmids at 1:3 ratio, 2 μg total plasmid). Cells were treated 24 h after transfection with either control (DMSO 0.1% (v/v)), 0.5 μM rapamycin, 2 μM everolimus or 50 nM wortmannin. The final DMSO concentration was kept under 0.1%. The cells were fixed 24 h after treatments with 4% PFA/PBS for 15 min and then washed in PBS, and coverslips were mounted with Mowiol 4-88 (Sigma-Aldrich).

#### Protein overexpression/knockdown

For analysis of the effect of protein overexpression, the cells were cotransfected with mGFP:mCherry plasmid Ras FRET pairs (DNA ratio 1:3) together with either 1.5 μg empty vector (control) or plasmids encoding Gal-1 or FKBP12 using JetPRIME reagent. For protein knockdown effect analysis, mGFP/mCherry plasmid FRET pairs (DNA ratio 1:3) were cotransfected with either scrambled siRNA (control), siRNA directed against FKBP12 (20 nM) or Gal-1 (50 nM) with JetPRIME reagent and fixation was performed 48 hours afterwards. SiRNA directed against PIK3CA (25 nM) were transfected with RNAiMAX (Thermo-Fischer Scientific, Grand Island, NY, USA) according to the manufacturer´s instructions. After 24 h of transfection, cells were transfected with mGFP/mCherry pairs, and after 48 hours cells were fixed with PFA and coverslips were mounted.

### FRET-imaging using fluorescence lifetime microscopy (FLIM)

The mGFP fluorescence lifetime was measured using a fluorescence lifetime imaging attachment (Lambert Instruments, Groningen, Netherlands) on an inverted microscope (Zeiss AXIO Ovserver.D1, Jena, Germany) [18]. For the sample excitation sinusoidally modulated 3 W, 497 nm LED at 40 MHz under epi-illumination was used. Cells were imaged using the 63x, NA 1.4 oil objective with the GFP filter set (excitation: BP 470/40, beam splitter: FT 495, emission: BP 525/50). The phase and modulation were determined using the manufacturer's software from images acquired at 12 phase settings. Fluorescein at 0.01 mM, pH 9.0 was used as a lifetime reference standard. For each treatment condition, the fluorescence lifetime was measured typically for >30 cells from three biological repeats. The percentage of the apparent FRET efficiency (E_app_) was calculated using the measured lifetimes of each donor-acceptor pair (τ_DA_) and the average lifetime of the donor only (τ_D_) samples. The formula employed was Eapp= (1-τ_DA_/τ_D_) x 100%.

### Confocal imaging

Confocal samples were prepared on glass bottom 10 mm microwell dishes (MatTek corporation, Ashland, MA, USA). Confocal images from live HEK cells were taken with a spinning disk confocal microscope (Marianas spinning disk imaging system with a Yokogawa CSU-W1 scanning unit on an inverted Carl Zeiss Axio Observer Z1 microscope, Intelligent Imaging Innovations, Inc., Denver, USA) using a 63x (NA 1.4 Oil, Plan-Apochromat, M27 with a DIC III Prism) objective. Images were treated using ImageJ 1.49n (National Institutes of Health, Bethesda, MD, USA) to adjust for contrast and to display them on gray-scale.

### Immuno-electron microscopic analysis of Ras nanoclustering

The detailed methodology of electron microscopic (EM) spatial mapping using the univariate K-function analysis can be found elsewhere [42, 43]. Briefly, the intact apical plasma membrane sheets of BHK cells expressing GFP-tagged constitutively active oncogenic Ras isoforms were attached to copper EM grids, which were fixed using 4% paraformaldehyde (PFA) and 0.1% glutaraldehyde. BHK apical plasma membrane sheets were immuno-labeled with 4.5 nm gold nanoparticles coupled to anti-GFP antibody, then embedded in uranyl acetate and imaged using TEM at 100,000X magnification. Gold particle x/y coordinates within a 1-μm^2^ area on intact and featureless plasma membrane sheets were assigned using ImageJ. Ripley's univariate K-function was used to quantify the lateral spatial distribution of gold particles (Eqs. A and B):

$$k(r)=\text{A}n^{-2}\sum {}_{i\neq j}w_{ij}1(\|x_{i}-x_{j}\|\leq r)$$ (Eq. A)

$$L(r)-r=\sqrt{\frac{k(r)}{\pi }}-r$$ (Eq. B)

where *K*(*r)* is the single population K-function for a lateral distribution of *n* points within the total plasma membrane area *A*; *r* indicates length scale with a range of 1 < *r* < 240 nm with 1 nm increments; ||.|| is Euclidean distance. The indicator function of 1(.) = 1 if ||*x_i_*-*x_j||_* ≤ r and 1(.) = 0 if ||*x_i_*-*x_j_* ||> r; and *w_ij_* ^−1^ is the circumferential fraction of the circle that has a center located at *x_i_* and radius ||*x_i_*-*x_j_* || To account for points at the edge of the study area, an unbiased edge correction was incorporated into the calculation. *K*(*r*) was then normalized to yield *L*(*r*) - *r* via using a 99% confidence interval (99% C.I.) estimated by Monte Carlo simulations. For each condition, at least 15 apical plasma membrane sheets were imaged, quantified and averaged to generate the shown K-function curves. A collection of 1000 bootstrap samples were constructed to evaluate the statistical significance between different conditions [44].

### PC12 cell differentiation

PC12 cells were plated in a 8-well Lab-Tek chambered coverglass (Nunc Thermo Fischer Scientific) coated with 0.1% rat tail collagen I in 30% ethanol. After 24 h, cells were transfected with GFP-H-rasG12V or GFP-K-rasG12V using Lipofectamine 3000 (Thermo- Fischer Scientific). Forty-eight hours after transfection, cells were treated for 3 days with either DMSO (control) or 0.5 μM rapamycin. GFP-expressing cells were imaged using an EVOS FL imaging system, and cells were scored for extension of neurites longer than 1.5 times the size of the cell soma. For each experimental condition, at least 100 GFP positive cells from four independent biological repeats were analyzed.

### *In ovo* tumor growth assay

Fertilized chicken eggs were placed in an egg incubator under rotation in 37°C and 60% humidity on day 1 of embryonic development. On day 3 the eggs were turned, taken off rotation and punctured with a small hole, then covered with adhesive tape. On day 8 the holes were expanded and a small plastic ring (5-6 mm in diameter) was placed on top of the chorioallantoic membrane (CAM). Subsequently, 2 x10^6^ MDA-MB-231 cells were suspended in a 1:1 mixture of PBS and matrigel for a total volume of 30 μl/egg and transplanted inside the plastic ring on the CAM, where after the eggs were sealed with parafilm. Tumors were treated daily either with control (DMSO 0.05% (v/v) in PBS) or 0.5 μM rapamycin in PBS. On day 12 the tumors were excised and fixed in 3% PFA overnight in + 4°C (or 1 hour at room temperature), after which the tumors were dehydrated with ethanol series of 50%, 70%, 70% for 1 h each at room temperature and finally 70% in + 4°C overnight. The tumors were weighed in 70% ethanol using an analytical laboratory scale.

### Sphere formation assay

Sphere formation assays were performed using HEK wildtype or transiently expressing mGFP-H-rasG12V or mGFP-K-rasG12V, MDA-MB-231 (K-rasG13D), Hs578T (H-rasG12D), and MCF7 (Ras wildtype) cells. For protein expression and knockdown experiments, cells were first seeded in 6-well plates and transfected with the indicated plasmids or siRNA with JetPRIME reagent for HEK and MCF7 cells, or with Lipofectamine 3000 reagent (Thermo Fisher Scientific) for MDA-MB-231 and Hs578T cells. 24 h later they were transferred to 48-well suspension culture plates (Cellstar®, Greiner Bio-One, Frickenhausen, Germany) at an initial density of 4,000 cells per well in serum-free media supplemented with 1x B27, 25 ng/ml EGF and 25 ng/ml FGF and grown for 9 days. For compound treatment experiments, 4,000 cells per well were plated in 48-well suspension culture plates. After 6 days in culture, cells were treated for 3 additional days with either control (DMSO 0.1% (v/v)), 0.5 μM rapamycin, 2 μM everolimus, 2 μM WYE-125132 or 0.25 μM torin 1. The spheres were analyzed in an Evos FL microscope (Thermo Fisher Scientific) and spheres with a size of at least 50 μm were counted. The sphere formation under different treatments was expressed as percentage normalized to the vehicle treated control. In the case of protein overexpression or knockdown samples, the samples were normalized to the empty vector or scrambled siRNA-transfected controls, respectively, and statistically compared.

### Immunoblotting

For SDS-PAGE analysis, cells were harvested in a buffer containing 50 mM dithiothreitol, 2% sodium dodecyl sulfate, 10% glycerol, 150 mM NaCl, 2 mM EDTA, 0.1 mM EGTA, 50 μg/ml PMSF, 0.1% bromophenol blue and 50 mM Tris-HCl, pH 6.8. Proteins were first separated using SDS polyacrylamide gels (10-12%) and then electroblotted on nitrocellulose membranes (Perkin Elmer, Waltham, MA, USA). Membranes were subjected to immunoblotting with the given antibodies and protein bands were detected with enhanced chemiluminescence (Clarity Western ECL Substrate, Bio-Rad, Helsinki, Finland) on a ChemiDoc MP system (Bio-Rad, Hercules, CA, USA). The protein bands were analysed with Image-Lab software (Bio-Rad).

### RNA-seq profiling

Briefly, 2D-cultured HEK cells untransfected (control) or transfected with FKBP12 siRNA or with a plasmid encoding rat Gal-1 or treated with 10 μM rapamycin for 6 h, 3D-cultured HEK cells and 2D- and 3D-cultured MDA-MB-231 cells were lysed and total RNA was extracted using the RNeasy Mini Kit (Qiagen). Quality of RNA samples was evaluated by the RNA Integrity Number (RIN) method. The samples were next prepared for the sequencing using Illumina TruSeq Stranded mRNA Sample Preparation Kit and they were sequenced with the HiSeq 3000 instrument using single-end sequencing chemistry and 50bp read length.

The gene-wise read count values from RNA-seq data were normalised using TMM normalisation as implemented in the edgeR R-package [45]. Prior to statistical testing, the data were further transformed using the voom approach in the limma R-package [46]. Differential expression analysis was then performed using the limma R-package, in which the moderate t-test was applied according to the specified contrasts (comparisons) between different sample groups. The statistical significance levels (p-values) were corrected for multiple hypotheses testing using false-discovery rate (FDR) approach as implemented in the limma R- package.

### Flow cytometric analysis of CD44+/CD24− cells

HEK cells were grown as spheres for 9 days under Gal-1 or FKBP12 level manipulation. Spheres were stained with APC-conjugated anti-CD44 (clone G44-26, BD Biosciences) antibody and PE-conjugated anti-CD24 antibody (clone ML5, BD Biosciences) following the manufacturer's instructions. Briefly, spheres were washed with phosphate-buffered saline and incubated in 0.05% trypsin/0.025% EDTA. Cells were next washed with phosphate-buffered saline containing 2% fetal calf serum and 0.1% sodium azide (FACS buffer 1) and resuspended in the FACS buffer 1 at 10^6^ cells/100 μl. Fluorochrome-conjugated monoclonal antibodies were added to the cell suspensions at concentrations recommended by the manufacturer and incubated at 4°C in the dark for 30–40 min. After staining, cells were washed with phosphate-buffered saline containing 0.1% sodium azide (FACS buffer 2), fixed using 4% PFA and cytometric analysis was performed in a FACS LSR II (BD Biosciences) cytometer in accordance with the manufacturer's protocols. In brief, unstained cells, CD44, CD24 and double-stained control cells were used to mark the four quadrants in a dot-plot for unstained, CD44+, CD24+ and double-positive populations. The change in percentage of CD44+/CD24− cells (top left quadrant) was measured in control and samples.

### Statistical analysis

Statistical analysis was used to support the main conclusions in this study. Unless otherwise specified, all experiments were performed at least three times. The sample size for each experiment is provided in the relevant figure legends and represents biological replicates/independent experiments performed on different days. Unless otherwise stated, statistical significance in Western blotting and *in-ovo* growth was evaluated with Student´s t-test. Statistical significance in the rest of the experiments was evaluated using an analysis of variance (ANOVA) complemented by Tukey's honest significance difference test (Tukey's HSD) performed in GraphPad PRISM software. Statistical significance levels are annotated as ns, not significant; *, p < 0.05; **, p < 0.01; ***, p < 0.001; ****, p < 0.0001.

## SUPPLEMENTARY MATERIALS FIGURES


