# Supplementary material for: Rapalogs can promote cancer cell stemness in vitro in a Galectin-1 and H-ras-dependent manner
Source: Oncotarget. 2017 May 11;8(27):44550–66. doi: 10.18632/oncotarget.17819 (PMC5546501; doi:10.18632/oncotarget.17819)
Supplement: Supplementary file 1 [file oncotarget-08-44550-s001.pdf]

Rapalogs can promote cancer cell stemness *in vitro* in a Galectin-1 and H-ras-dependent manner

SUPPLEMENTARY MATERIALS

SUPPLEMENTARY FIGURES

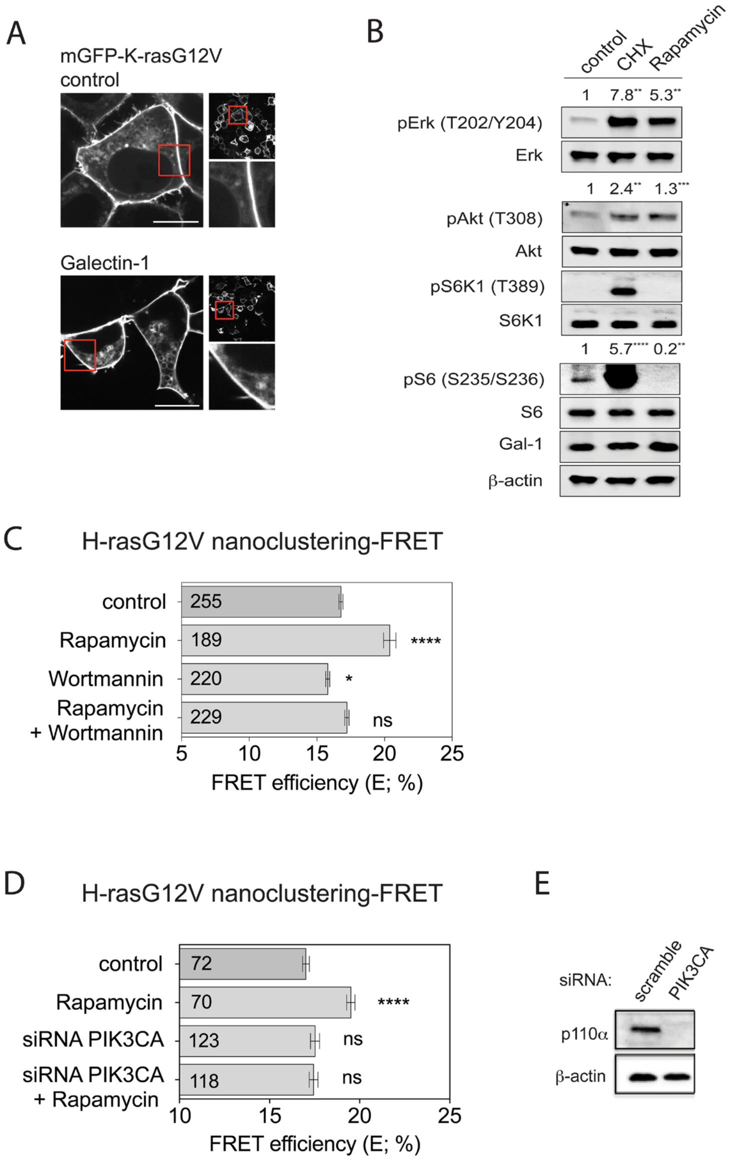

Supplementary Figure 1: Rapamycin increases H-ras-nanoclustering in a PI3K-dependent manner. (A) Subcellular localization of mGFP-K-rasG12V expressed in HEK cells (*top*) or co-expressed with Gal-1 (*bottom*). Representative images from two independent experiments are shown. Scale bar in the images represents 10 µm. **(B)** Western blot analysis of Ras- and mTORC1-signaling in BHK21 cells treated for 24 h with either DMSO control, 0.18 µM CHX or 0.5 µM rapamycin. Numbers indicate the ratio of phosphorylated to respective total protein levels (n=3). **(C)** Nanoclustering-FRET analysis in HEK cells coexpressing mGFP- and mCherry-tagged H-rasG12V. Cells were treated for 24 h with DMSO control, 0.5 µM rapamycin, 50 nM wortmannin or both. The numbers in the bars indicate the number of analyzed cells (mean ± SEM, n≥3). **(D)** Nanoclustering-FRET analysis in HEK cells transfected with scrambled siRNA or siRNA directed against the PIK3CA gene product (i.e. PI3K p110α), co-expressing mGFP- and mCherry-tagged H-rasG12V. Cells were treated for 24 h with either DMSO control or 0.5 µM rapamycin. The numbers in the bars indicate the number of analyzed cells (mean ± SEM, n≥3). **(E)** Western blot analysis of PI3K p110α knockdown in HEK cells expressing mGFP-H-rasG12V.

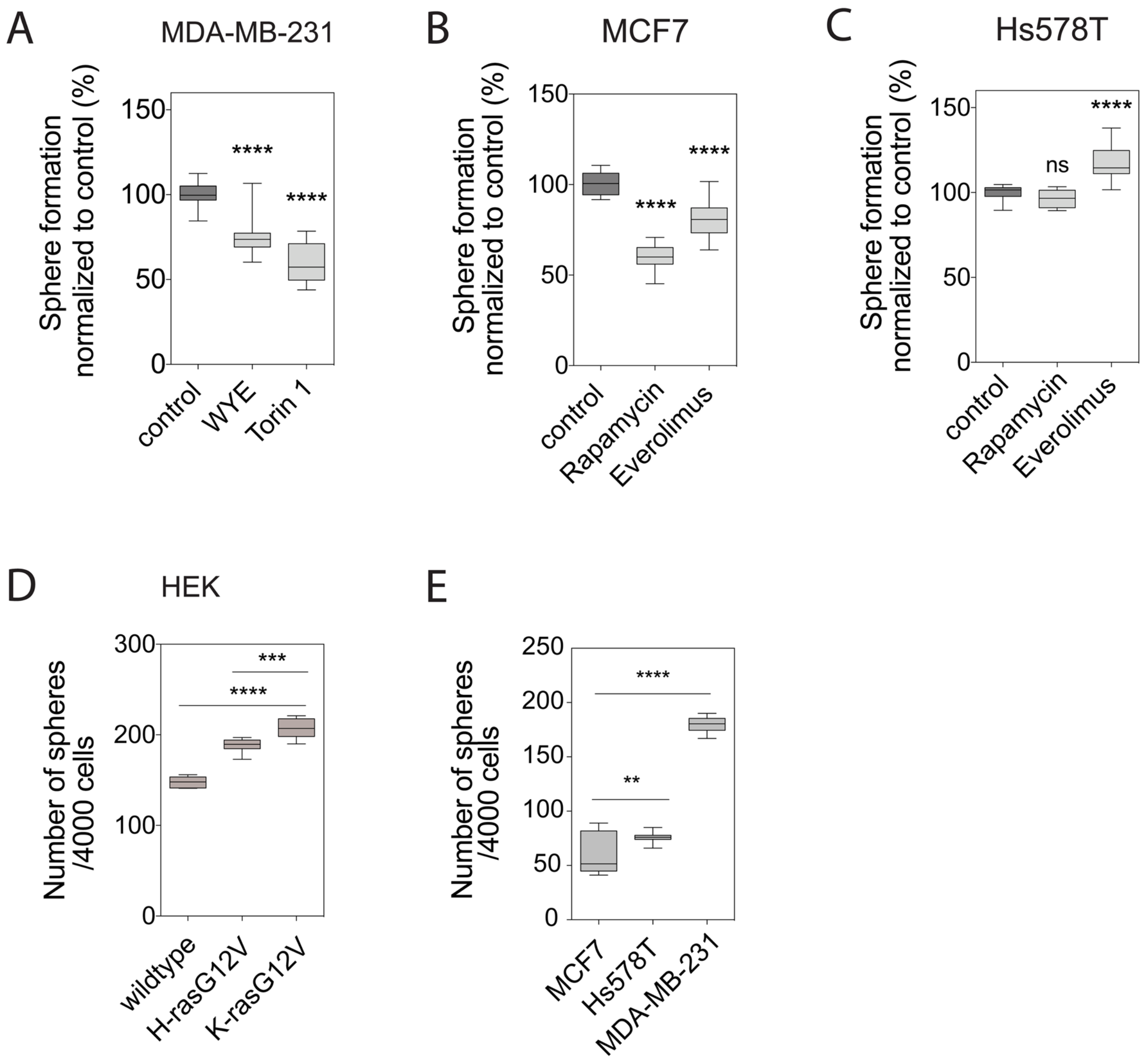

Supplementary Figure 2: Rapalogs and mTor-catalytic inhibitors impact on Ras-dependent tumorosphere formation. (**A** to **C**) Mammosphere-forming efficiency of **(A)** MDA-MB-231, **(B)** MCF7, **(C)** Hs578T cells grown under non-adherent conditions. Mammospheres were allowed to form for 6 days and treated for additional 3 days with either DMSO control, 2 µM WYE-125132, 0.25 µM torin 1, 0.5 µM rapamycin or 2 µM everolimus (n=3).(**D** and **E**) Absolute numbers of spheres of the indicated cell lines. HEK cells transiently expressed indicated Ras constructs.

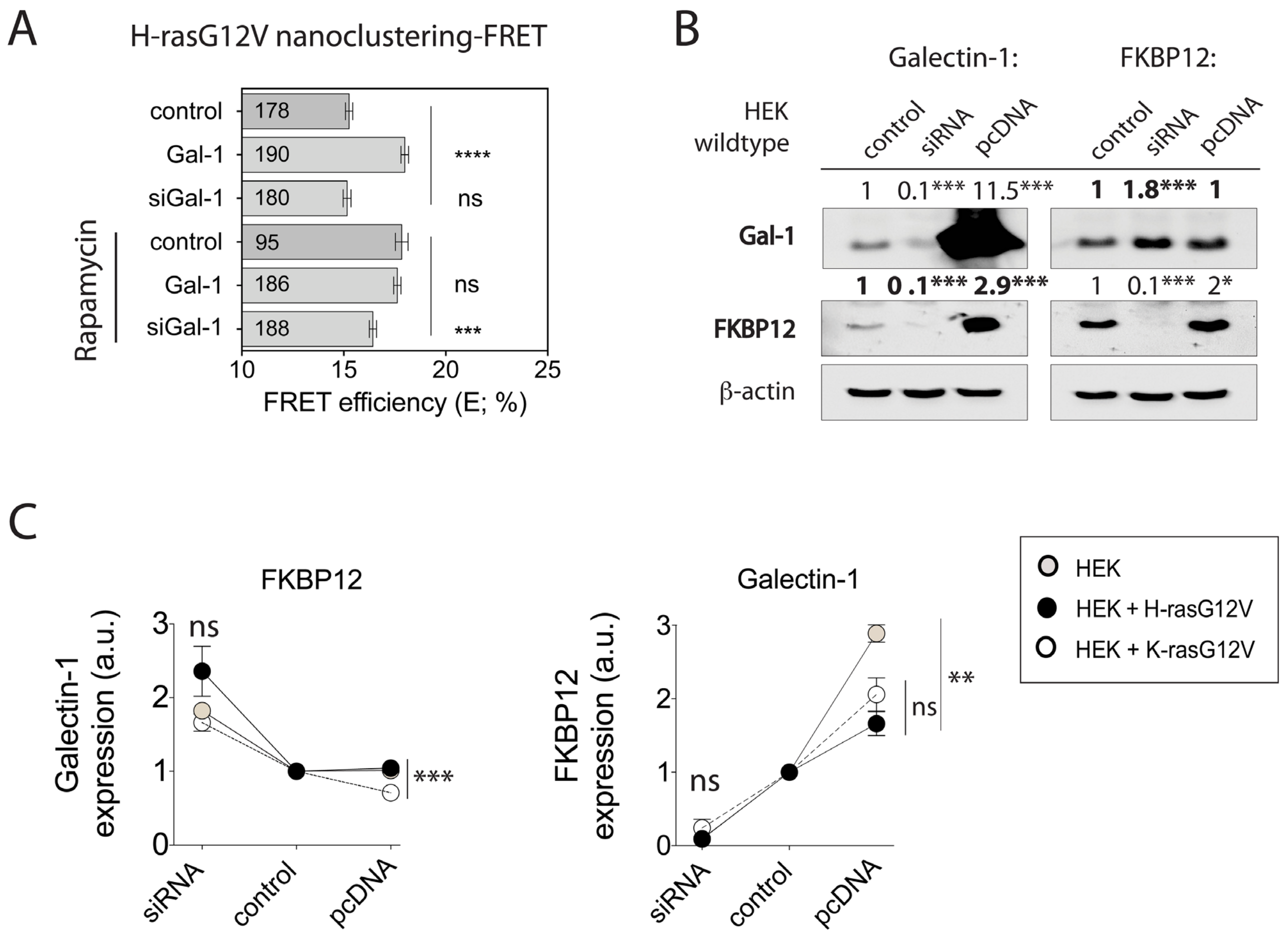

Supplementary Figure 3: Galectin-1 and FKBP12 expression is coupled. (A) Nanoclustering-FRET analysis in galectin-1 knockdown/overexpressing HEK cells coexpressing mGFP- and mCherry-tagged H-rasG12V. Cells were treated for 24 h with either DMSO control or 0.5 µM rapamycin. The numbers in the bars indicate the number of analyzed cells (mean ± SEM, n=3). **(B)** Western blot analysis of Gal-1/FKBP12 expression level crosstalk in wildtype HEK cells. Numbers indicate β-actin normalized protein levels (n=4). **(C)** Summary of quantitative analysis from (Figure 3C, 3D and Supplementary Figure 3B) (mean ± SEM). Statistical significance was examined using 2-way ANOVA.

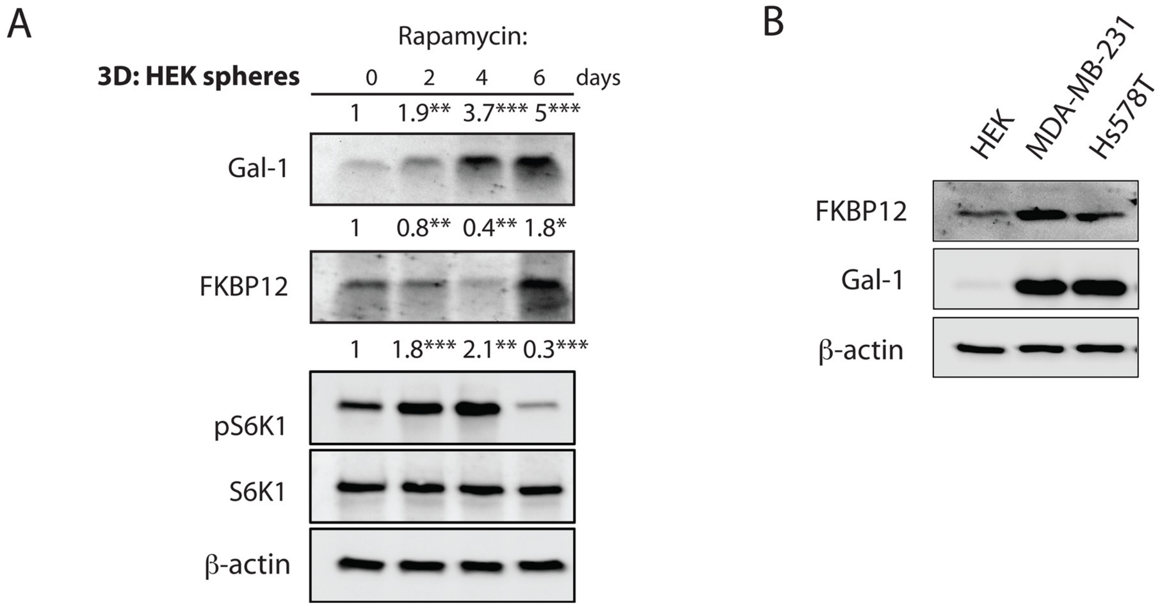

Supplementary Figure 4: FKBP12-binding inhibitors induce downmodulation of FKBP12 thus increasing galectin-1 expression. (A) Western blot analysis of Gal-1 and FKBP12 protein levels in wildtype HEK spheres upon 0.5 µM rapamycin treatment for 6 days. Numbers indicate β-actin normalized protein levels. pS6K1 is shown as a marker of mTORC1 activity, and numbers indicate the ratio of phosphorylated to respective total protein levels (n=3). **(B)** Western blot analysis of endogenous Gal-1 and FKBP12 protein levels in HEK, MDA-MB-231 and Hs578T.
